# Age- and Sex-Dependent Heterogeneity in Mumps Virus Immunity Among US Blood Donors Born Between 1928 and 1993

**DOI:** 10.1093/infdis/jiag379

**Published:** 2026-07-19

**Authors:** Aum R. Patel, Axel A. Guzmán-Solís, Gregory A. Foster, David Krysztof, Susan L. Stramer, Benhur Lee, Jean K. Lim

**Affiliations:** 1 Department of Microbiology, Icahn School of Medicine at Mount Sinai, New York, New York, USA; 2 Graduate School of Biomedical Sciences, Icahn School of Medicine at Mount Sinai, New York, New York, USA; 3 Scientific Support Office, Transfusion Safety & Surveillance, American Red Cross, Rockville, Maryland, USA

**Keywords:** mumps, seroprevalence, neutralization, immunity, MMR

## Abstract

**Background.:**

Despite widespread vaccination, mumps outbreaks continue to occur, raising concerns about waning immunity and strain-specific differences in neutralizing antibody responses.

**Methods.:**

We evaluated seroprevalence, total antibody levels, and neutralization capacity against the vaccine (JL5) and outbreak (genotype G) mumps virus (MuV) strains from 187 US blood donors born between 1928 and 1993. Associations with age, sex, and donation site, were analyzed.

**Results.:**

MuV IgG seropositivity was 84.5% and did not differ by age, sex, or blood donation site. However, ELISA index values increased with age, with individuals ≥60 years exhibiting higher total MuV antibody levels than donors ≤30 years, primarily driven by males. For most donors, neutralization was consistently higher against JL5 than genotype G, and ELISA index values positively correlated with neutralization titers for both strains. Age positively associated with genotype G neutralization but not JL5, with a significant increase in the genotype G:JL5 neutralization ratio with age (*P* < .0001). Males exhibited significantly higher neutralization titers than females against both strains, with older males exhibiting greater neutralization against Genotype G than both age-matched females and younger males.

**Conclusions.:**

Our study suggests that antibody quantity alone does not fully predict mumps protection, and factors such as sex may contribute to functional differences in immunity. Our findings highlight potential gaps in protection against circulating strains, possibly contributing to ongoing outbreaks despite high vaccination coverage. The age-dependent increase in total MuV antibodies, which correlates with genotype G neutralization, supports that cumulative antigenic exposure may shape the breadth of MuV-specific immunity over time.

Mumps virus (MuV), a member of the genus *Orthorubulavirus* within the *Paramyxoviridae* family, remains an important vaccine-preventable pathogen despite the availability of effective live-attenuated vaccines. Before the vaccine era, infection was nearly universal in childhood, with seropositivity exceeding 90% by adolescence [1–3], driven by efficient respiratory transmission (R_0_ up to ~14) [4]. Following the introduction of the measles-mumps-rubella (MMR) vaccine in 1971, incidence declined dramatically in the United States (US) and other countries [2, 5]. Nevertheless, since 2000, outbreaks have re-emerged in countries with historically high vaccine coverage, particularly among young adults living in close-contact settings, including individuals fully vaccinated [3, 6, 7]. These patterns have raised important questions regarding the durability, quality, and breadth of vaccine-induced immunity.

Protection against MuV is generally thought to depend, in part, on neutralizing antibodies against the viral surface antigens, primarily hemagglutinin-neuraminidase and fusion (F) proteins [8], although correlates of protection remain incompletely defined [9]. While MuV-specific IgG seropositivity is widely used as a marker of immunity, binding antibodies do not always predict neutralization or protection from infection [10, 11]. Indeed, several studies have suggested vaccinated or seropositive individuals can still become infected, suggesting that qualitative aspects of the antibody response, rather than quantity alone, may also be critical determinants of protection [7, 12].

A central concern in ongoing mumps outbreaks is the potential mismatch between vaccine strains and circulating viruses. Current vaccines are derived from genotype A strains (Jeryl Lynn lineage), whereas most contemporary outbreaks are caused by genotype G viruses [11]. Although MuV is considered serologically monotypic, accumulating evidence suggests that antigenic differences between genotypes may reduce cross-neutralization, potentially contributing to breakthrough infections [4, 11, 13].

In parallel, waning immunity has also been proposed as a contributor to resurgence [7, 14], with studies showing increased susceptibility in adolescents and young adults vaccinated in early childhood, consistent with declining antibody quantity or function over time [9]. The interplay between age, vaccination status, and infection severity/outcomes can be broken down into 2 premises: (1) age-related severity (regardless of vaccination) and (2) vaccination effect within age groups. Irrespective of vaccination status, there are heightened rates of complications in MuV-infected young adults compared with children [15, 16]. Secondly, despite waning immunity, vaccinated individuals are better protected against severe mumps disease. This is supported by historically lower rates of hospitalization and complications, lower virus isolation rates, and likely shorter duration of viral detection compared with unvaccinated individuals [12, 15–18], underscoring continued vaccine effectiveness.

In the United States, MuV serostatus is primarily assessed by enzyme-linked immunosorbent assay (ELISA), which provides a qualitative measure of antibody reactivity to the vaccine strain [19, 20]. While useful for screening, this approach does not capture functional neutralization or define protective thresholds [9]. Currently, the relationship between antibody quantity, functional activity, and demographic variables such as age and sex remains incompletely understood, including how repeated exposure or prevaccine infection history may shape cross-strain immunity. Among younger individuals whose mumps immunity originated from childhood vaccination, repeated natural exposures to circulating virus, either symptomatic or subclinical, may act to boost and diversify the antibody repertoire, potentially enhancing cross-strain neutralization over time [21]. Among older individuals born prior to the widespread implementation of mumps vaccination, natural exposure may have resulted in higher or more durable total antibody levels and superior cross-strain neutralization. In this study, we characterized MuV immunity in US blood donors by measuring ELISA-based seropositivity and total IgG levels by ELISA and evaluated neutralization against the vaccine strain (JL5) and a contemporary genotype G strain, integrating these data with demographic variables, including age and sex, to define patterns of immune heterogeneity.

## METHODS

### Study Participants

This study utilized plasma from the American Red Cross (ARC) blood donations collected between 2003 and 2011 that were removed from the US blood supply due to initial reactivity on West Nile virus (WNV) nucleic acid amplification testing, as previously described [22]. Samples were derived from 2 donor cohorts comprising 1255 blood donations. The first cohort (2003–2008) included 1099 donations, of which 143 were classified as WNV-negative. All 143 WNV-negative samples from this cohort were included in the present study. To achieve balanced representation across age and sex, an additional 44 samples were selected from a second cohort of 156 donations collected between 2008 and 2011 without knowledge of WNV status; these were subsequently identified as 10 WNV-negative and 34 WNV-positive donors. In total, 187 donors were analyzed. All donors self-identified as White, Non-Hispanic, a sociocultural designation that does not reflect biological diversity. Donor demographics are summarized in Table 1. Plasma samples were heat-inactivated at 56°C for 1 hour, clarified by centrifugation, and stored at 4°C until use.

### Mumps Virus ELISA

Total mumps-reactive antibodies were measured using a commercially available mumps IgG ELISA kit (Arlington Scientific), which utilizes whole cell lysates from Jeryl Lynn-5 (JL5)-infected Vero cells, following manufacturer protocols. Plasma samples were diluted 1:51 and added to antigen-coated wells along with calibrator controls for standard curve generation. Absorbance was measured at 405 nm using a Cytation 3 plate reader, and ELISA index values were calculated from a standard curve. Per manufacturer protocol, samples with index values >1.1 were considered seropositive, <0.9 as seronegative, and 0.9–1.1 as equivocal. Equivocal samples were analyzed separately and subsequently grouped with seronegative samples for the study.

### Mumps Virus Amplification and Rescue

Vaccine strain, Jeryl Lynn-5 (MuV-JL5) was generated from a plasmid encoding the MuV-JL5 antigenome (gift from W. Paul Duprex). The construct included a codon-optimized T7 promoter and hammerhead ribozyme at the 3′ end and a hepatitis delta virus ribozyme at the 5′ end to drive efficient rescue. For viral rescue, BSR-T7 cells were transfected with antigenomic MuV-JL5 plasmid (5 μg), T7-JL5-N (0.3 μg), T7-JL5-P (0.1 μg), T7-JL5-L (0.2 μg), optimized T7 polymerase (2 μg), PLUS reagent (7.5μL), and Lipofectamine LTX (18.75μL) in OptiMEM [22]. Virus was amplified on Vero cells, clarified, pelleted through a 20% sucrose cushion by ultracentrifugation (24 000 RPM for 3 hours), resuspended in RPMI, and stored at −80°C. The clinical MuV genotype G strain (Iowa 2006) was provided by Bettina Bankamp (CDC) and propagated and stored using the same procedure.

### Mumps Virus Plaque Assay

Vero cells were maintained in DMEM with 10% fetal bovine serum (FBS), 1% penicillin-streptomycin, and 1% L-glutamine (DMEM growth media). Cells were seeded in 24-well plates and infected the following day with serially diluted virus in infection media (DMEM with 2% FBS, 1% Penicillin-Streptomycin, 1% L-glutamine) for 1 hour at 37°C with periodic agitation. Cells were then overlaid with 1% methylcellulose in infection media and incubated for 72 hours. Cells were then fixed with 4% paraformaldehyde (PFA), stained with hyperimmune guinea pig serum (1:1000) and then Alexa Fluor-488-conjugated antiguinea pig secondary (1:1000) in intracellular staining buffer (0.1% saponin, 0.2% BSA) and imaged using the Celigo S platform.

### Mumps Virus Neutralization Assay

Plasma samples were serially diluted in RPMI supplemented with 10% FBS, 1% penicillin-streptomycin, and 1% L-Glutamine, incubated with virus for 1 hour at 37°C, and used to infect Jurkat T cells at an MOI of 1.25. After 24 hours, cells were fixed with 2% PFA, permeabilized in PBS containing 0.2% BSA, 0.1% saponin, and stained with anti-MuV-F monoclonal antibody (Sigma; MAB846; 1:5000) followed by a goat anti-mouse IgG antibody (Thermo Fisher; P-852). Cells were acquired on the Attune NxT Flow Cytometer and analyzed using FlowJo v10.8.2.

### Statistical Analysis

Differences between 2 groups were assessed using a Student’s *t*-test for continuous variables, and a Fisher’s exact test for categorical outcomes. Continuous outcomes (ELISA indices) were analyzed using linear regression. Age, sex, and other covariates were first evaluated in univariate analyses, including assessment of outliers, transformations where appropriate, and exploration of potential confounding and interaction effects. Variables with *P* < 0.15 in univariate analyses were entered into multivariable linear models. *P* values were not adjusted for multiple comparisons. For all statistical analysis, data was analyzed using the SAS System software (SAS Institute, Inc., Cary, NC).

## RESULTS

### Seroprevalence of Mumps Virus Antibodies Among US Blood Donors

To assess population-level MuV immunity in the US, we analyzed plasma from 187 blood donors from the ARC. The cohort was balanced by sex (50.3% male, 49.7% female) and age (16–80 years; median, 48 years), and recruited from rural (15.5%), suburban (31%), and urban (53.5%) donation sites (Table 1). Mumps virus serostatus was determined independent of vaccination history by ELISA, the standard assay for assessing MuV immunity. This assay detects total MuV-reactive antibodies using crude lysates from vaccine strain (JL5)-infected cells but does not differentiate between neutralizing and non-neutralizing responses. Index values were calculated for each sample and classified as seropositive (≥1.1), seronegative (≤0.9), or equivocal (0.9–1.1); equivocal samples were grouped with seronegative individuals. Overall, 84.5% (158/187) of donors were seropositive, whereas 15.5% were seronegative (Table 1). Seropositivity did not differ significantly by sex, age groups or donation site (Table 1), indicating broadly consistent MuV serostatus across demographic strata.

### Total Mumps Virus Antibody Levels Positively Correlate With Age

Having established overall seropositivity among US blood donors, we next examined whether total MuV-specific antibody levels, measured by ELISA index values, varied by demographic characteristics using a multivariate analysis incorporating age, sex, and donation site. Because routine MMR vaccination began in the early 1970s, we next compared antibody levels between individuals born prior to (prevaccine era) and after (vaccine era) 1972. Those born in the prevaccine era exhibited significantly higher antibody levels than those in the vaccine era, consistent with more durable immunity following natural infection and/or repeated exposure (Figure 1A). Stratifying donors by age revealed a progressive increase in ELISA index values with age, reaching significance in individuals ≥60 years compared with those ≤30 years (mean 4.11 vs 2.44, *P* = 0.004), while intermediate age groups showed similar but nonsignificant trends (Figure 1B and Table 2). Consistent with these findings, seronegativity was lowest among individuals ≥60 years of age (4.5%) compared with the younger age groups, where seronegativity ranged between 17% and 23% (Table 1). Neither sex nor donation site was independently associated with antibody levels; however, the age-related increase was primarily driven by older males (Figure 1C–D) Together, these findings indicate that older males exhibit the highest MuV-specific antibody levels, while other demographic factors have little measurable effect.

### Mumps Virus Antibodies Display Reduced Neutralization of Genotype G

While ELISA provides a useful measure of total MuV-specific antibodies, it does not distinguish between neutralizing and non-neutralizing responses and therefore cannot directly assess protective immunity. To determine whether antibody magnitude corresponded to functional activity, we evaluated the neutralization capacity of plasma from 187 blood donors. Plasma samples were serially diluted and incubated with either the MuV vaccine strain (JL5) or a contemporary outbreak-associated genotype G strain prior to infection of Jurkat T cells. After 24 hours, infection was quantified by intracellular staining for viral F antigen followed by flow cytometric analysis, and IC50 values were calculated relative to infection in the absence of plasma.

Given the absence of a defined correlate of protection for MuV, we next assessed the relationship between total antibody levels and neutralization capacity. To do this, we compared mean reciprocal IC50 across donors and found that values were substantially higher for JL5 compared with genotype G (166.99 vs 59.65; Table 2, Figure 2A), indicating reduced functional antibody activity against the circulating strain. Log-transformed reciprocal IC50 values demonstrated a moderate positive correlation with ELISA index values for both JL5 (*r*^2^ = 0.3336) and genotype G (*r*^2^ = 0.3395) (Figure 2B–C), suggesting that higher antibody levels are generally associated with increased neutralizing activity. However, this relationship was imperfect. Notably, despite being classified as seropositive by ELISA, a subset of individuals lacked detectable neutralizing activity, including 1 individual against JL5 and 8 against genotype G.

### Age- and Sex-Dependent Differences in Neutralization Capacity and Breadth

Given these findings, we next evaluated whether neutralization titers varied according to demographic factors. Although sex was not associated with differences in seropositivity or total antibody levels by ELISA, multivariable analysis revealed a significant effect of sex on functional antibody responses (Table 2). Specifically, males exhibited higher neutralization titers than females against both JL5 (179.16 vs 154.68, *P* = 0.026) and genotype G (70.20 vs 48.98, *P* = 0.025). No significant differences in neutralization capacity were observed across blood collection sites.

To more directly assess the relationship between antibody quantity and functional breadth, we normalized neutralization capacity by calculating the ratio of genotype G to JL5 IC50 values for each individual. This approach accounts for inter-individual differences in total antibody levels and enables comparison of relative neutralization efficiency across strains. As shown in Figure 3A, this ratio exhibited a positive correlation with age (*r*^2^ = 0.0931; *P* < 0.0001), indicating that older individuals retain relatively greater neutralization capacity against the heterologous genotype G strain compared with younger donors.

In contrast to total antibody index values, neutralization capacity revealed an interesting pattern of age-dependency. JL5 neutralization titers were comparable across ages, with no significant differences observed across prevaccine or vaccine era as well as age brackets for males and females (Figure 3B–C and Table 2). However, genotype G neutralization capacity increased with age, with individuals aged 46–59 demonstrating significantly higher titers compared with those ≤30 years (IC50 = 91.11 vs 44.58, *P* = 0.012), with a similar trend observed relative to the ≥60 group that did not reach statistical significance (*P* = 0.076; Table 2). This effect appears to be driven by prevaccine era males, as they demonstrated a significantly higher capacity to neutralize genotype G than both prevaccine females and vaccine era males (Figure 3D–E).

## DISCUSSION

Prior to vaccination, MuV infection was nearly universal in childhood, with seropositivity exceeding 90% by adulthood [1]. The introduction of the MMR vaccine sharply reduced disease incidence, yet outbreaks have re-emerged since the early 2000s in highly vaccinated populations, with a notable epidemiologic shift toward young adults [4, 23, 24]. These observations raise concerns regarding the durability, quality, and breadth of vaccine-induced immunity. In this study, we assessed binding and neutralizing antibody responses in US blood donors and identified substantial immune heterogeneity to MuV not captured by standard serologic approaches.

Several key findings emerge. Although overall seroprevalence remains high at 84.5%, this level is below the approximately 90% threshold estimated for herd immunity required to prevent sustained transmission of a highly contagious virus such as MuV [4, 14]. Especially concerning are recent trends in vaccine hesitancy and increasing measles and mumps outbreaks, which suggests that herd immunity levels are likely even lower [25]. These gaps are particularly evident in younger individuals, where seropositivity rates are lower and total antibody levels are reduced compared with older age groups. In contrast, individuals ≥60 years of age showed the highest antibody levels and lowest seronegativity rates, consistent with durable immunity following natural infection or repeated exposure. While vaccination has reduced cases by >95% [2], waning immunity in younger, vaccine-derived cohorts appears to contribute to susceptibility, consistent with epidemiologic patterns in high-density settings, such as universities and military environments [14]. However, antibody quantity alone did not fully explain susceptibility patterns.

A central finding of this study is the marked reduction in neutralizing activity against a contemporary genotype G strain compared with the vaccine strain JL5. Across the cohort, neutralization of genotype G was approximately threefold lower, despite the presence of detectable binding antibodies. This provides direct functional evidence supporting the hypothesis that antigenic differences between vaccine and circulating strains contribute to incomplete protection and consistent with several other studies [10, 11, 13]. Although MuV has traditionally been considered serologically monotypic [13], our data add to accumulating evidence that neutralization efficiency varies across genotypes [26]. At the population level, even modest reductions in neutralization capacity may have meaningful consequences for transmission dynamics, particularly in settings with high contact rates.

Importantly, a subset of individuals classified as seropositive lacked detectable neutralizing activity, particularly against genotype G. This discordance underscores a key limitation of current diagnostic paradigms, which rely heavily on ELISA-based serostatus as a surrogate for immunity. In the case of MuV, the commercially available ELISA relies on antigen derived from JL-infected Vero cells, which no longer circulates and is known to have antigenic differences in its surface epitopes as compared with Genotype G [11]. These findings indicate that seropositivity alone may overestimate functional immunity and suggest that qualitative features of the antibody response, such as specificity, affinity, or epitope targeting, may differentially influence neutralization capacity, particularly against currently circulating strains. Furthermore, binding assays detect total antibody reactivity but do not discriminate between neutralizing and non-neutralizing responses, nor do they account for strain-specific differences in antigenicity.

Demographic analyses further revealed that functional antibody responses are shaped by both sex and age. While sex was not associated with differences in seropositivity or total antibody levels, males exhibited significantly higher neutralization titers against both JL5 and genotype G. This finding contrasts with the broader literature in viral immunology, where females often mount stronger humoral responses, and suggests that host-intrinsic factors may influence the quality of the antibody response following infection or vaccination between sexes [27–32]. Supporting this observation, prior outbreak data have reported higher attack rates among females [33, 34], raising the possibility that reduced neutralization capacity may contribute to increased susceptibility. The underlying mechanisms remain unclear but may involve hormonal regulation of B cell responses, differences in immune activation, or variation in exposure history [28, 32]. Larger, more diverse studies will be required to confirm and mechanistically dissect these sex-specific effects.

Age-related differences in neutralization capacity were more nuanced. Neutralization of the vaccine strain JL5 was relatively stable across age groups, indicating that homologous immunity is largely preserved. In contrast, neutralization of genotype G exhibited greater variability, with middle-aged and older individuals demonstrating improved responses compared with younger donors. This pattern was further clarified by normalizing genotype G neutralization to JL5 responses, revealing an age-associated increase in neutralization breadth. These findings suggest that older individuals possess not only higher antibody levels but also a more functionally diverse antibody repertoire capable of recognizing heterologous strains.

Together, these data support a model in which immune history critically shapes both the magnitude and quality of the antibody response. Individuals born prior to vaccine implementation were likely exposed to wild-type MuV and may have experienced repeated boosting through adult-vaccination in addition to natural or subclinical infection. Importantly, while Genotype A was the predominant circulating strain during the prevaccine era, the older cohort in our study still demonstrated superior cross-neutralization of genotype G. This suggests that immune imprinting, or “Original Antigenic Sin” from initial Genotype A exposure, does not permanently restrict the antibody repertoire. Rather, lifelong cumulative exposures may promote affinity maturation and the development of antibodies targeting conserved epitopes, thereby enhancing cross-strain neutralization. In contrast, vaccine-induced immunity, while sufficient to generate seropositivity, may be more narrowly focused and less capable of accommodating antigenic variation, especially given its current composition (JL5/JL2). As the proportion of individuals with natural immunity declines over time, population-level immunity may become increasingly dependent on vaccine-induced responses that are less durable and less broad. These findings have important implications for vaccine strategy. While current MMR vaccines remain highly effective at preventing severe disease, they may be less effective at preventing infection and transmission, particularly in the context of antigenically divergent strains [9]. Although modeling studies suggest that waning immunity is a primary driver of outbreaks, our data indicate that strain-specific differences in neutralization may further exacerbate susceptibility. This raises the possibility that updated vaccine formulations incorporating contemporary genotypes, or strategies aimed at enhancing antibody breadth and durability, could improve population-level protection.

Several limitations should be considered. The cohort was restricted to self-identified White, non-Hispanic individuals, limiting generalizability to more diverse populations. Age brackets were also intentionally balanced rather than reflecting the US population distribution, which may affect interpretation of age-related differences, particularly since antibody levels and neutralization titers tend to be higher in older adults. In addition, donors represent a generally healthy, healthcare-accessible population and may not reflect individuals with limited access to care. Another limitation is that samples were selected from blood donations initially flagged for suspected WNV positivity and are not truly random blood donors. While only a small subset of samples was WNV seropositive, we do not believe that WNV infection itself lead to a biased sample distribution. Further, vaccination and infection histories were unavailable, precluding direct assessment of exposure-specific immunity. immune responses. Finally, in vitro neutralization does not capture the full complexity of in vivo immunity, including cellular responses and mucosal immunity. Despite these limitations, the consistency of the observed relationships across multiple analyses strengthens the overall conclusions.

In summary, this study demonstrates that MuV immunity in the United States is characterized by significant quantitative and functional heterogeneity. Seropositivity alone is an incomplete measure of protection, as neutralization varies across individuals, is influenced by age and sex, and is reduced against genotype G compared with the vaccine strain. This disconnect between binding and functional immunity provides a plausible explanation for ongoing outbreaks in vaccinated populations. Although waning immunity is well established, our data suggest that the breadth and durability of MuV-specific immunity reflect cumulative antigenic exposure from vaccination and/or natural infection. Defining correlates of protection that incorporate both antibody magnitude and function will be essential. These findings highlight the need to reassess current serological approaches to assessing immunity and to develop strategies that improve both durability and breadth of vaccine-induced responses.

## Supplementary Material

Supplemental Material

## Figures and Tables

**Figure 1.**
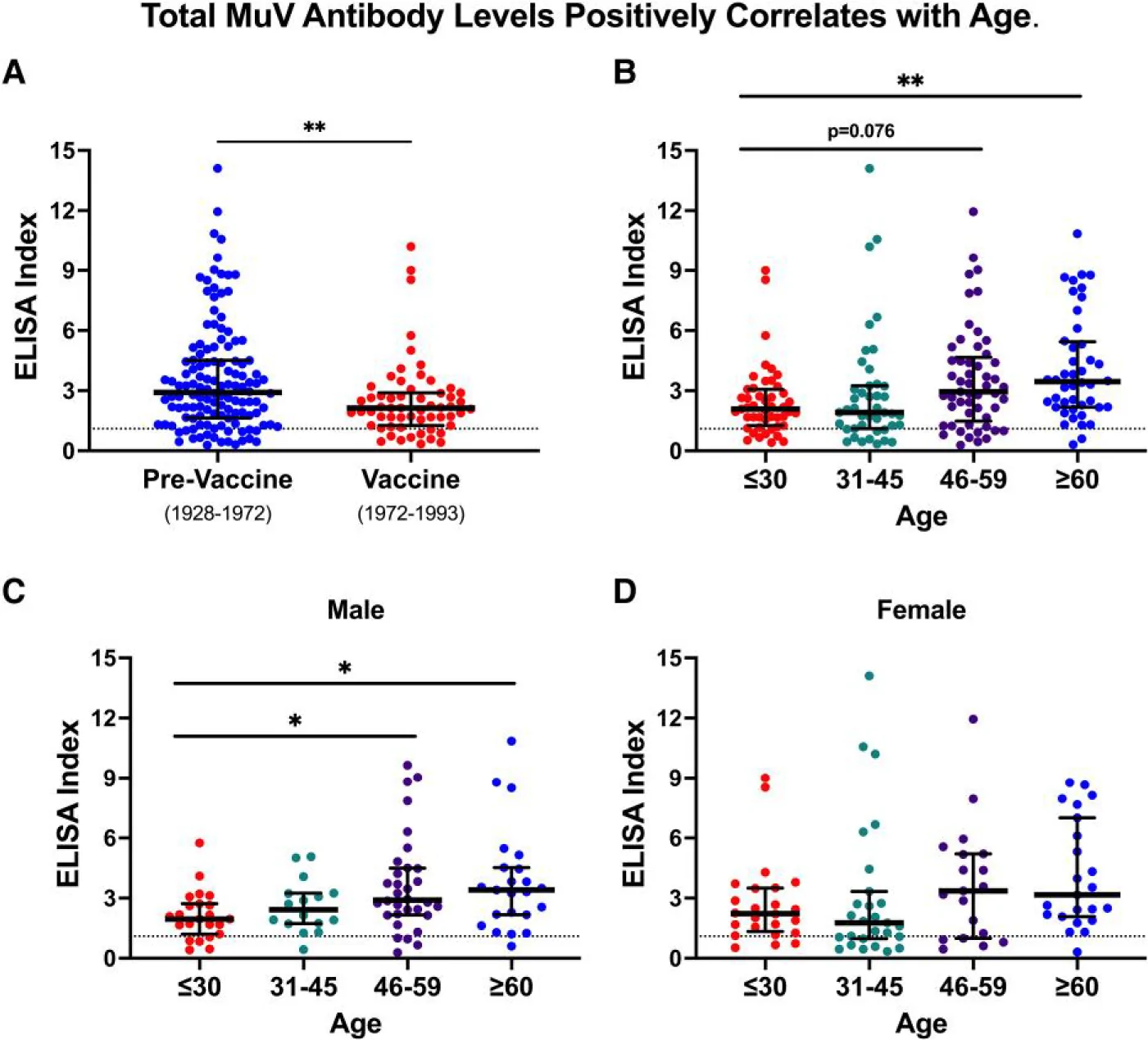
Total MuV antibody levels positively correlate with age. All donors were evaluated for total mumps-reactive antibodies by ELISA. *A*, Comparison of MuV ELISA index values between donors born before widespread MMR implementation (prevaccine; before 1972) and those born in the vaccine era (vaccine; after 1972). *B*, Index values were stratified by age group as indicated. Age analyses were separately performed for either males (*C*) or females (*D*). Horizontal bars represent median ± IQR. Kruskal-Wallis test was performed for all panels Significant values are denoted by *P ≤ .05; **P ≤ .01.

**Figure 2.**
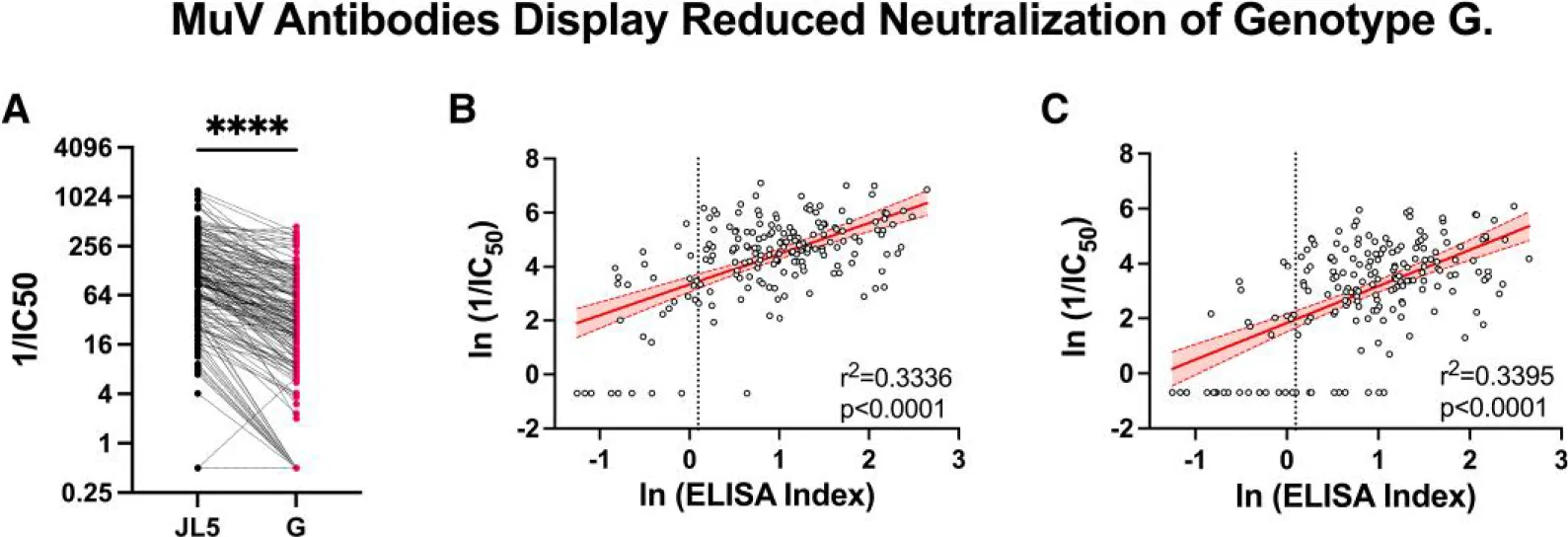
MuV antibodies display reduced neutralization of genotype G. Neutralization assays were performed for JL5 and genotype G to calculate IC50 values for individual blood donors; donor blood that was unable to neutralize virus were given an IC50 values of 0.5. *A*, Reciprocal IC50 values for each donor are plotted for JL5 and G, with lines connecting individual donors. ELISA index values and reciprocal IC50 were plotted using natural log transformation for JL5 (*B*) and genotype G (*C*) correlation analysis. Vertical line indicates an index value of ln(1.1), demarcating seronegative (left) and seropositive (right) individuals by ELISA. For statistical analysis, a paired t-test was used for (*A*). For (*B–C*) a simple linear regression was used. ****P ≤ .0001.

**Figure 3.**
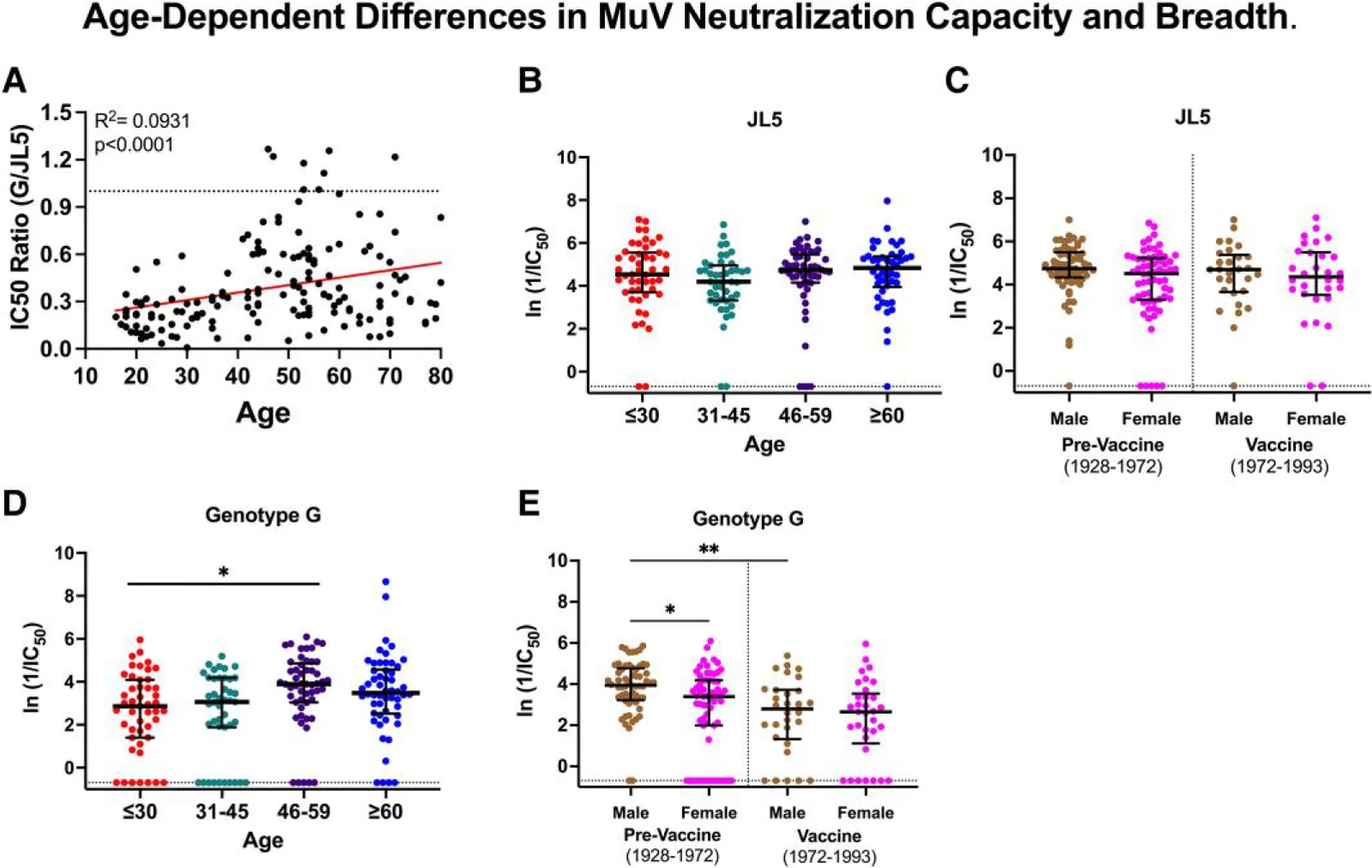
Age-dependent differences in MuV neutralization capacity and breadth. *A*, Neutralization titers, expressed as reciprocal IC50 values following natural log transformation against JL5 or genotype G. A ratio was then calculated of Genotype G:JL and plotted against age. MuV strains, were stratified across age groups. Neutralization titers against JL5 were then analyzed by age bracket (*B*) or placement into the prevaccine or vaccine era and analyzed by sex (*C*). Similar analyses were performed for Genotype G based on age (*D*) and sex (*E*). Individuals without a measurable IC50 were eliminated. For statistical analysis multiple linear regressions were used. Each point represents an individual donor, and horizontal bars denote median ± IQR. For (*A*), a simple linear regression was performed. For (*B–E*), a Kruskal-Wallis test was performed. Significant values are denoted by *P ≤ .05; **P ≤ .01.

**Table 1.** Mumps Virus Seroprevalence Among US Blood Donors

	N (%)	Seropositive (%)	Seronegative (%)	OR	95% CI	*P* Value

Total	187 (100%)	158 (84.5%)	29 (15.5%)	…	…	
Sex	…	…	…	…	…	
Female	93 (49.7%)	75 (80.6%)	18 (19.4%)	1.81	0.79–3.89	0.1625
Male	94 (50.3%)	83 (88.3%)	11 (11.7%)	…	…	
Age	…	…	…	…	…	
≤30	47 (25.1%)	39 (83.0%)	8 (17.0%)	-	-	-
31–45	43 (23.0%)	33 (76.7%)	10 (23.3%)	0.70	0.24–2.05	0.5156
46–59	53 (28.3%)	44 (83.0%)	9 (17.0%)	0.84	0.28–2.49	0.7534
≥60	44 (23.5%)	42 (95.5%)	2 (4.5%)	4.06	0.80–20.70	0.0921
Donation site	…	…	…	…	…	
Suburban	58 (31.0%)	45 (77.6%)	13 (22.4%)	-	-	-
Rural	29 (15.5%)	27 (93.1%)	2 (6.9%)	4.02	0.81–20.0	0.1787
Urban	100 (53.5%)	86 (86.0%)	14 (14.0%)	1.97	0.82–4.75	0.9726

**Table 2.** Mumps Virus Antibody Binding and Neutralization Among US Blood Donors

		ELISA			JL5 Neutralization (1/IC50 Values)			Genotype G Neutralization (1/IC50 Values)		
	N (%)	Mean (SD)	Median	*P* Value	Mean (SD)	Median	*P* Value	Mean (SD)	Median	*P* Value

Total	187 (100%)	3.26 (2.55)	2.61		166.99 (202.54)	105.22	-	59.65 (80.94)	29.71	-
Sex	…	…	…	…		…	…	…	…	…
Female	93 (49.7%)	3.38 (2.89)	2.45	-	154.68 (209.25)	89.85	-	48.98 (75.69)	23.32	-
Male	94 (50.3%)	3.14 (2.16)	2.69	0.734	179.16 (196.03)	112.66	0.**026**	70.20 (84.91)	37.61	0.**025**
Age	…	…	…	…		…	…	…	…	…
≤30	47 (25.1%)	2.44 (1.75)	2.09	-	208.49 (271.90)	92.68	-	44.58 (70.56)	17.5	-
31–45	43 (23.0%)	2.89 (2.90)	1.92	0.962	123.98 (172.29)	66.09	0.348	36.71 (42.18)	21.37	0.911
46–59	53 (28.3%)	3.58 (2.58)	2.95	0.076	168.90 (181.30)	113.29	0.691	91.11 (106.37)	49.12	0.**012**
≥60	44 (23.5%)	4.11 (2.61)	3.45	0.**004**	162.38 (160.79)	114.21	0.935	60.25 (74.83)	32.57	0.076
Collection Site		…	…	…	…		…	…	…	…
Suburban	58 (31.0%)	3.12 (2.72)	2.5	-	167.85 (175.82)	115.21	-	67.50 (89.13)	30.1	-
Rural	29 (15.5%)	3.91 (2.59)	3.55	0.096	118.05 (87.41)	105.22	0.381	36.66 (30.08)	31.46	0.968
Urban	100 (53.5%)	3.16 (2.43)	2.54	0.272	180.67 (237.11)	97.85	0.19	61.76 (85.35)	28.86	0.892

## Data Availability

The data in this article are available as supplementary online material.
